# Testing Accuracy of Long-Range Ultrasonic Sensors for Olive Tree Canopy Measurements

**DOI:** 10.3390/s150202902

**Published:** 2015-01-28

**Authors:** Juan Luis Gamarra-Diezma, Antonio Miranda-Fuentes, Jordi Llorens, Andrés Cuenca, Gregorio L. Blanco-Roldán, Antonio Rodríguez-Lizana

**Affiliations:** 1 Dpto. de Ingeniería Rural, Área de Mecanización y Tecnología Rural, Universidad de Córdoba, Córdoba 14005, Spain; E-Mails: o02gadij@uco.es (J.L.G.-D.); ir2llcaj@uco.es (J.L.); ir2cucua@uco.es (A.C.); ir3blrog@uco.es (G.L.B.-R.); 2 Dpto. de Ingeniería Aeroespacial y Mecánica de Fluidos, Área de Ingeniería Agroforestal, Universidad de Sevilla, Ctra. de Utrera, km 1. 41013 Sevilla, Spain; E-Mail: arodriguez2@us.es

**Keywords:** ultrasonic sensor, canopy characterization, olive tree, sensor accuracy, sensor interference

## Abstract

Ultrasonic sensors are often used to adjust spray volume by allowing the calculation of the crown volume of tree crops. The special conditions of the olive tree require the use of long-range sensors, which are less accurate and faster than the most commonly used sensors. The main objectives of the study were to determine the suitability of the sensor in terms of sound cone determination, angle errors, crosstalk errors and field measurements. Different laboratory tests were performed to check the suitability of a commercial long-range ultrasonic sensor, as were the experimental determination of the sound cone diameter at several distances for several target materials, the determination of the influence of the angle of incidence of the sound wave on the target and distance on the accuracy of measurements for several materials and the determination of the importance of the errors due to interference between sensors for different sensor spacings and distances for two different materials. Furthermore, sensor accuracy was tested under real field conditions. The results show that the studied sensor is appropriate for olive trees because the sound cone is narrower for an olive tree than for the other studied materials, the olive tree canopy does not have a large influence on the sensor accuracy with respect to distance and angle, the interference errors are insignificant for high sensor spacings and the sensor's field distance measurements were deemed sufficiently accurate.

## 1. Introduction

Olives are a key crop along the Mediterranean basin and, more specifically, in Spain, which is the main olive oil producer in the world, with a cultivated area in 2013 of 2.6 Mha (15% of the cultivated area in that country) [1]. This crop presents a low level of new applied technologies and a huge variety of plantation types and patterns with three basic cultivation systems: traditional, intensive, and super-intensive or hedgerow olive plantations [2].

Crop protection with phytosanitary products is, still today, a barely used technology. In order to optimize application volumes, it is necessary to obtain real-time information about the geometrical characteristics of the target vegetation to adjust the pesticide dose [3]. Furthermore, the European directive (128/2009/CE) for the sustainable use of pesticides [4] establishes the dose adjustment and reduction as a key requirement to achieve high-quality food production.

In this sense, the main commercial system currently available is the ultrasonic sensor, which allows an air blast sprayer to spray exclusively when vegetation is detected. Its use for olives is limited to automatically opening electrovalves when an object is detected within its measurement range, assuming it to be the target canopy. Because these sensors allow for the measurement of distances, they have been used in several studies to determine the dimensions or volumes of tree crops [5–8]. In this sense, ultrasonic sensors have advantages and disadvantages. On the one hand, they are cheap, robust [9], and very easy to use [10]. In addition, they have exhibited a reasonable accuracy under field conditions, which is sufficient for most cases [11]. On the other hand, their main drawback is the error produced by some factors, mainly the shape and distance to the target and the interference with the signal from the next sensors. Furthermore, the bare information obtained by each sensor results in a very low spatial resolution, requiring the use of a larger number of sensors to cover a real target [12].

Regarding the interference, errors occur because of sonic cone widening. If two sensors are too close, there is a high probability that crosstalk occurs between them with an inherent accuracy loss. In the literature, this has been overcome in two ways: (i) by vertically separating the sensors until the errors become insignificant and (ii) by using more sensors and synchronizing the readings of groups of sensors [11].

Ultrasonic sensor measurements have been widely used for various applications to different crops such as vineyards [10,13–15], orchard fruits [16–18], or citrus [11,19–21]. Thus, the canopy volume to be sprayed by a group of nozzles was calculated from the distances obtained by the sensors, allowing for real-time regulation of the pesticide dose to be sprayed on the different rows. The sensors' measurements from the center of the sprayer track are complementary to the tree width, and the volume can be calculated by multiplying for the manually-measured tree height. Different algorithms for tree volume calculation were employed in the aforementioned works. For this type of application, it is necessary to ensure that the sensor lectures are sufficiently accurate, and different authors have performed studies to determine the accuracy of ultrasonic sensors under different operational conditions or by accounting for different variables.

Specifically, Tumbo *et al*. [11] carried out a trial to assess the accuracy of an ultrasonic sensor by comparing its measurements with manual measurements of crown volumes and with a laser sensor for citrus. They observed a very good correlation between the volumes calculated from the sensor measurements and the manually measured volumes, but they detected a small overestimation of the volumes calculated from the ultrasonic sensor's measurements. A similar result was obtained by Zaman and Schumann [22], who compared the volumes measured manually and by ultrasonic sensors in orange tree fields with different ages and tree spacings. In this case, the volumes measured by ultrasonic sensors were overestimated with respect to the manually measured volumes as well, although statistically significant differences were not observed. Another conclusion was that the tree age and spacing have a negative effect on the measurement accuracy; therefore, the authors recommend increasing the sampling frequency in those cases.

Llorens *et al*. [10] compared manual measurements with those obtained from ultrasonic and a Light Detection And Ranging (LiDAR) sensors in vineyards for different phenological states. More specifically, Escolà *et al*. [23] tested a commercial sensor, a Sonar Bero PXS400 M30 K3 (Siemens AG, Munich, Germany). They used six sensors mounted on a vertical mast to measure distances in an apple tree plantation, and they determined their accuracy by comparing with manual measurements, obtaining a mean error of ±5.11 cm. They tested the interference between two sensors as well by separating them at distances of 30 and 60 cm, and they obtained errors of ±17.46 cm and ±9.39 cm, respectively. Therefore, it was concluded that 60 cm is the minimum sensor spacing for operation under field conditions. Jeon *et al*. [16] exposed the sensor to different adverse climatic conditions such as extreme temperatures, dust, and a cross wind. They found that some of these factors can produce an increase in the RMSE up to ±5 cm, as in the case of high-temperature exposure.

For the most common olive tree type in Spain, the row spacing very frequently reaches 12 m in the traditional cultivation system with a cultivated area of 1.85 Mha, which is 76% of the total olive area [2]. This means that the ultrasonic sensors used for other crops are not able to operate in this area owing to the insufficient measurement range. Therefore, sensors with a higher measurement range are required for olives (up to 6 m), but this type of sensor has some drawbacks, with the lower sampling frequency and wider sound cone being the most important. As stated earlier, Zaman and Schumann [22] recommend increasing the sampling frequency for old and widely spaced trees (as is the case for traditional olive trees). These circumstances require a detailed study of the long-range sensors to determine its suitability for field conditions.

In this study, the main objective is to determine the suitability of a long-range ultrasonic sensor (model UC6000-30GM-IUR2-V15, Pepperl + Fuchs, Mannheim, Germany) for use in olive tree canopies. Four partial objectives are defined:

- To determine the sensor's sound cone widening for three targets: olive tree canopies, an aluminum panel, and a reference plastic surface with dimensions of 100 × 100 mm.
- To assess the measurement errors as a consequence of the incident angle of a sound wave for four different materials with varying rugosity: an olive tree canopy, a rough wall, a corkboard, and a smooth surface.
- To determine the errors produced by the crosstalk between sensors for multiple sensor spacings in an olive canopy and a rough wall at different distances.
- To assess the validity of the sensor calibration obtained in the laboratory for field measurements and determine the sensor's accuracy in these conditions and for different distances.

## 2. Materials and Methods

### 2.1. Sensor, Control Unit, and System Connections

Traditional olives are frequently planted at row distances of 12 m; therefore, a sensor that can measure distances up to 6 m was selected. The selected sensor was the Pepperl + Fuchs UC6000-30GM-IUR2-V15, and its main characteristics are listed in Table 1. This sensor requires a power supply of 24 V_DC_, and it provides a dual analog output as current or voltage. In the default configuration, this sensor provides a current of 4 to 20 mA. To measure with a wide range, the sensor's accuracy is slightly reduced, especially when widening the sound cone, which is wider than that of a lower range sensor. Even though its data acquisition is relatively slow, the sensor has very appropriate characteristics for field work, such as a high degree of protection and resistance to vibrations.

A programmable automation controller (PAC; model CompactRIO 9025, National Instruments, Austin, TX, USA; its technical specifications are listed in Table 2) was used as control unit. A power supply of 24 V_DC_/5 A (Phoenix Contact, Blomberg, Germany) was used for the CompactRIO PAC and sensor to stabilize the signal noise because the presence of voltage peaks could damage the devices. The sensor was connected to the CompactRIO PAC by an analog-signal acquisition module (NI 9203, National Instruments). Data were visualized and stored in a laptop computer. The specific software for system management and data acquisition was developed for the trial in LabView (National Instruments), the same platform used to control the computer.

### 2.2. Sensor Calibration

In order to measure distances with the sensor is necessary to calibrate it by obtaining the relationship between the measured distance and the analog output (current in units of mA). To perform the test, the sensor was placed at different distances from a smooth, uniform aluminum board, and analog signal value was read and stored for every distance. By using the stored results, a calibration function was fitted by the least-squares method:

(1) Real distance=a×Intensity−b

### 2.3. Experimental Determination of the Sound Lecture Cone

According to the specifications from the manufacturer, the widening cone of the sound waves emitted by the sensor markedly varies depending on the target [24]. As the main purpose of the study was to measure the distances to vegetation, a trial was planned to determine the sound cone with an olive crown acting as a target. In order to determine the variation of the sonic cone with different target materials and, therefore, the sensor measurements homogeneity, three different targets were used: a 1 × 1 m aluminum board; a plastic square surface with dimensions of 100 × 100 mm, as described by the manufacturer in the data sheet to serve as a reference; and a small-sized olive tree. The 100 × 100 mm surface was selected to determine if the trial conditions were similar to the manufacturer's.

**Figure 1.**
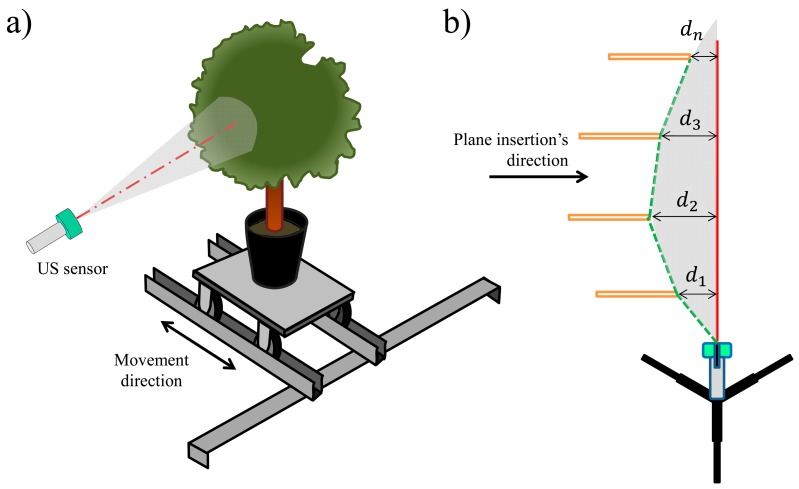
Methodology used to determine the lecture cone of the ultrasonic sensor. (**a**) Perspective view (**b**) Top view.

To perform the trial, a wheeled platform was used (Figure 1) and moved along two steel UPE profiles (European Standard U Profiles) placed perpendicular to the sensor's longitudinal axis and screwed into two fixed supports. A visible laser emitter was placed parallel to the longitudinal axis of the sensor to indicate the exact measurement point. The test consisted of moving the platform closer to the longitudinal axis of the sensor and monitoring its signal in real-time. When the interface of the CompactRIO PAC exhibited a change in the lecture (the moment at which the sensor detected a target on the platform), a laser telemeter (GLM 50 Professional, BOSCH, Chicago, IL, USA) with a precision of ±1.5 mm was used to measure the distance between the axis of the sensor and the most external part of the target perpendicular to the sensor's longitudinal axis. The test was repeated every 25 cm from 1 to 6 m, with three repetitions per position.

### 2.4. Determination of the Influence of the Reflection Angle on the Measurement Accuracy

As the working principle of the sensor lies in the quantification of the elapsed time between the emission and reception of a sound wave that impacts a target, it is logical to conclude that the reflection angle on that target will affect the accuracy of the measurements. Analogously, it can be concluded that the rugosity of the target will be another factor to account for. The effect of the reflection angle on the measurement accuracy could be very important for isolated trees because they do not always remain perpendicular to the sensor when obtaining measurements along the row.

**Figure 2.**
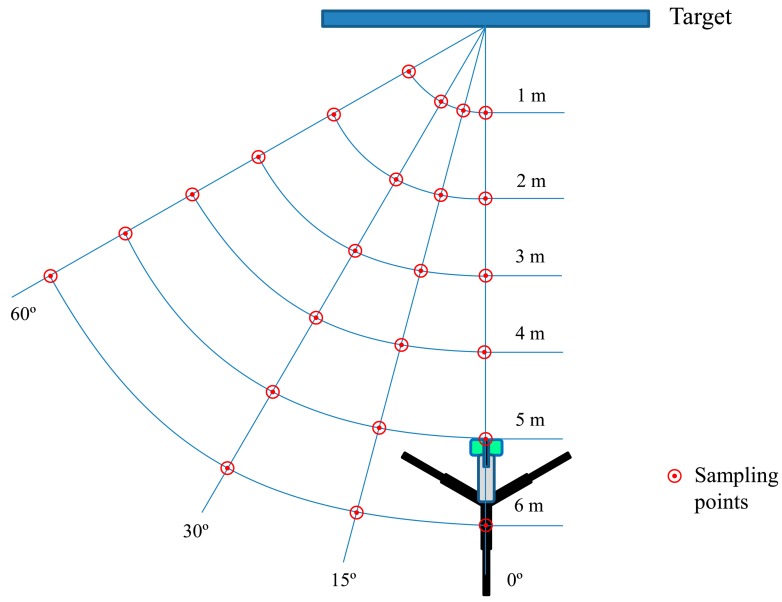
Top view of field arrangement of the sensor positions for measurements in the reflection angle effect trial.

Therefore, a trial was established to assess the absolute and relative errors of the sensor measurements respect to the actual manually measured distances. Four different angles (0°, 15°, 30°, and 60°), six distances (1, 2, 3, 4, 5, and 6 m), and four materials with different rugosity (a smooth plastic board with dimensions of 1 × 1 m, a corkboard with dimensions of 1 × 1 m, a rough wall, and an olive tree) were selected (Figure 2). Thirty readings were obtained for each position (Sampling zone on Figure 2) at a frequency of 1 Hz.

To evaluate the incident reading angle for every selected material, a model and lineal regression with artificial variables (*Z_1_, Z_2_, Z_3_*) were fitted, including their multiplicative effects:

(2) $$d_{{\text{measured}}_{i}}=\gamma_{0}d_{{\text{real}}_{i}}+\sum_{k=1}^{3}\gamma_{k}Z_{k_{i}}d_{{\text{real}}_{i}}+{ɛ}_{i}$$

where *d_measured_* is the measured distance to the target by the sensor; *d_real_* is the real, manually measured distance to the target; and ε*_i_* is the random error. The significance level of the coefficients γ_1_, γ_2_, and γ_3_ for every different material is obtained to determine if there is any variation in the relationship between the real and measured distances as a consequence of the reflection angle.

### 2.5. Determination of the Effect of Crosstalk between Sensors

**Figure 3.**
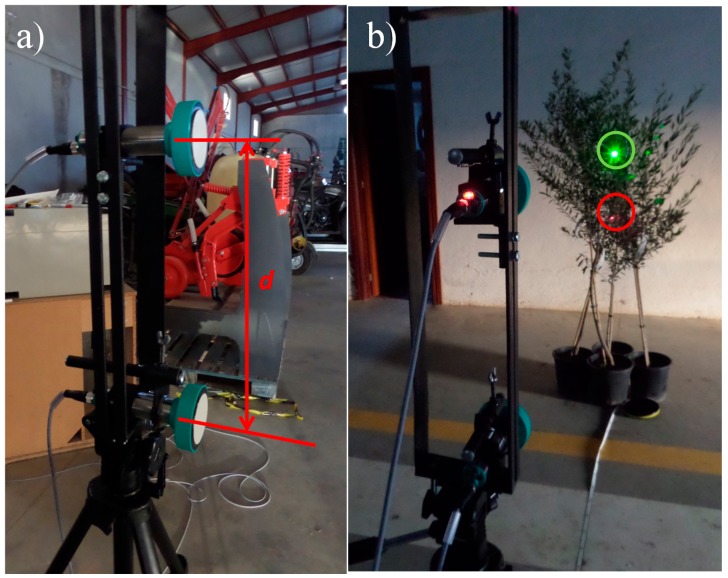
(**a**) Sensor mounting for evaluating measurement interference as a function of the sensor spacing *d*; (**b**) Laser beams on the target canopy.

The most usual arrangement for ultrasonic sensors is on vertical masts [13,16]. In this system, the dispersion of a sonic wave could generate interference in the measurements taken by sensors placed next. Therefore, a trial was established to quantify the errors produced as a consequence of the use of simultaneous readings of an ultrasonic sensor in the measurements taken by another ultrasonic sensor following a similar methodology described elsewhere [23]. Sensors were mounted on a mast especially designed for the trial, and the sensor spacing (*d* in Figure 3a), target distance, and target material were varied.

Two different targets (a rough wall and an olive tree canopy), eight sensor spacings (30, 40, 50, 60, 70, 80, 90, and 100 cm, plus 130 cm only for the olive tree target), and six target distances (1, 2, 3, 4, 5, and 6 m) were tested with 30 readings per combination with a measurement frequency of 1 Hz. In order to measure the distance between the sensor and the target, two laser beams were oriented to be parallel to the longitudinal axis of the two sensors used (Figure 3b). Measurements were performed with the laser telemeter by pointing at an artificial plane target set at the most external part of the canopy. The interference of every sensor at each position was calculated as the difference between the mean of the measurements when measuring alone and the mean of measurements when reading simultaneously with the other sensor. With the results obtained when interference is present, an interpolation by ordinary kriging was generated as a function of the target distance and sensor spacing. For both materials, the olive tree and the rough wall, a spherical variogram model with geometric anisotropy was used [25], being the main directions those from the coordinate axes.

### 2.6. Determination of the Accuracy of the Sensor under Field Conditions

**Figure 4.**
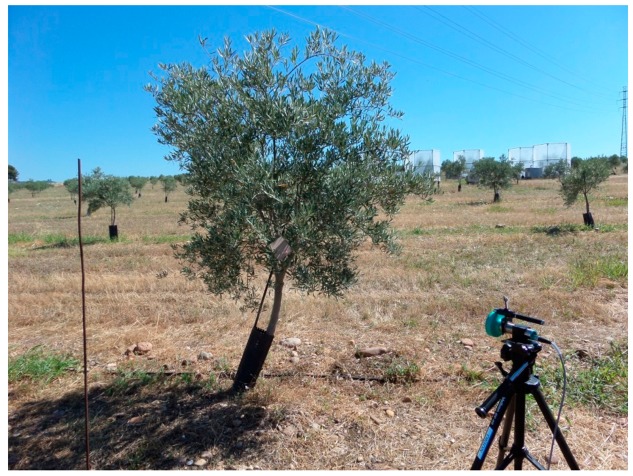
Positioning of the sensor for distance measurements in the field.

The next step was to test the sensor under real field conditions. The field of study was the Campus of Rabanales (University of Córdoba) (37°55′15.12”N; 4°43′15.58”W), which has olive trees of the varieties Picual, Arbequina, Hojiblanca, Manzanilla and Gordal and of a short age. The tree spacing is 6 × 6 m, and the mean canopy volume was 0.79 m^3^. A single sensor was tested, and the distances to the tree canopies were measured from different points that were randomly selected in a way similar to that in [23]. The measurement methodology was the following: (a) acquisition of 30 measurements with the ultrasonic sensor placed on an adjustable leveled tripod (Figure 4); (b) repetition of the measurements with a square target of 100 × 100 mm at a point perpendicular to the sensor (indicated by a laser beam); and (c) measurement of the real distance to the target with the laser telemeter. A percentage unitary coefficient of reading similarity (s_l_) was then calculated by dividing the mean measurement without the square target by the mean measurement with the square target. Very low values of the coefficient indicated some error in the corresponding reading. A total of 50 different positions were tested for different trees within the field of study with a total of 60 readings per position (30 with and 30 without the target). The goal of this trial is to determine the sensor's accuracy when measuring under real field conditions.

## 3. Results and Discussion

### 3.1. Calibration Function of the Sensor

During calibration of the sensor, data were fitted to the following linear model:

(3) $$\text{Real distance}\left(\text{cm}\right)=32319.80\times \text{Intensity}\left(\text{A}\right)-49.30,{\text{R}}^{2}=0.999,p<10^{-5}$$

This function made it possible to work with distances, which is much more intuitive, in the rest of trials performed. The calibration test showed that the sensor presents a “blind” distance at which it is unable to obtain any measurement, which is mentioned in the specifications of the manufacturer. It was also found that there is another distance range at which measurements taken by the sensor are not correct. In this case, it is possible to configure the width of this range, which is from 40 to 80 cm in the default configuration.

### 3.2. Cone Definition

**Figure 5.**
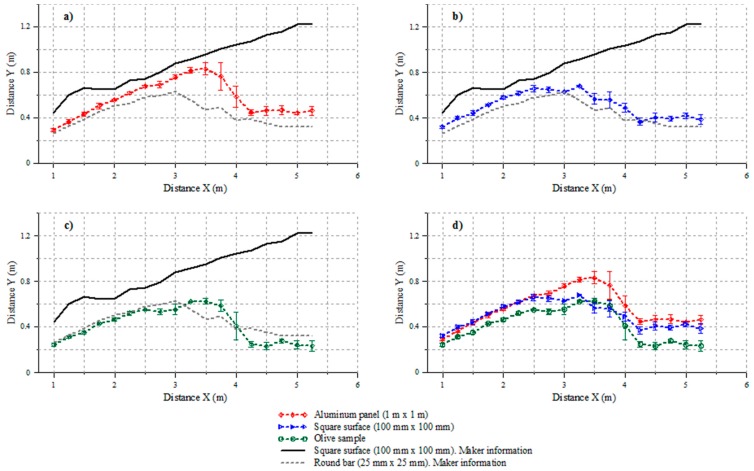
Results for the cone widths (only half of the cone is represented) at every distance compared to the manufacturer's specifications given by the sensor's datasheet: (**a**) an 1 × 1 m aluminum board; (**b**) a 100 × 100 mm standard square target; (**c**) an olive tree canopy; and (**d**) all studied materials together.

The results obtained for the cone shape for the different tested materials are shown in Figure 5. From the results obtained for the different tested materials, it is found that the sensor exhibits different behavior compared to that indicated by the manufacturer (Figure 5), even when one of the targets has the same shape and dimensions (Figure 5b). According to the manufacturer, the sonic cone exhibits continuous widening within the studied range up to 5.25 m, whereas all of the studied materials in this trial exhibit a maximum at 3.5 m for the aluminum board and at 3.25 m for the square target and olive canopy.

In addition, the data describe a narrower cone than that specified by the manufacturer for the 100 × 100 mm square target for every distance and material, which may cause technicians to avoid the use of this sensor for certain applications when it would be appropriate. For example, for the 100 × 100 mm square target, a cone diameter of 2.4 m is expected at a distance of 5 m according to the manufacturer [24]. The present study reveals that the real diameter for that distance is 0.8 m (33% of the value indicated by the manufacturer).

The definition of the sound cone results in much more uncertainty in the zone where it attains a maximum. This result, already observed in the trial, was confirmed by the larger typical errors observed for distances between 3 and 4 m, resulting lower for short and long distances in general. In all cases, the cone becomes narrower for distances greater than 4 m, but its increments and decrements are not the same abrupt in all the cases.

The narrowest cone is observed for the olive tree (Figure 5d), with the exception of distances in the range of 3.5–3.75 m. This fact could be explained by the larger absorption of sonic energy with this material. This absorption could be due to the porosity present in the vegetation, which reduces the number of reflections that can take place in the most external zones; therefore, the returning sound wave would only be at most perpendicularly reflected.

A possible explanation for the difference between the results obtained in the present study and by the manufacturer could be due to the differences in the trial conditions, *i.e.*, even if the same target was used, the rest of the parameters present in the manufacturer's trial are not specified in the sensor's datasheet. Taking into account traditional olive tree spacings and dimensions, the distances at which the sensor would more frequently operate would be the distance range of 2–5.5 m. This means that the maximum cone width, placed at 3.5 m distance from the sensor, is within the range of distances frequently read by the sensor. The expected cone width will range between 46 cm (±5 cm) for a 5.25-m distance and 1.2 m (±2 cm) for a 3.5-m distance.

### 3.3. Angle Effect Trial

**Figure 6.**
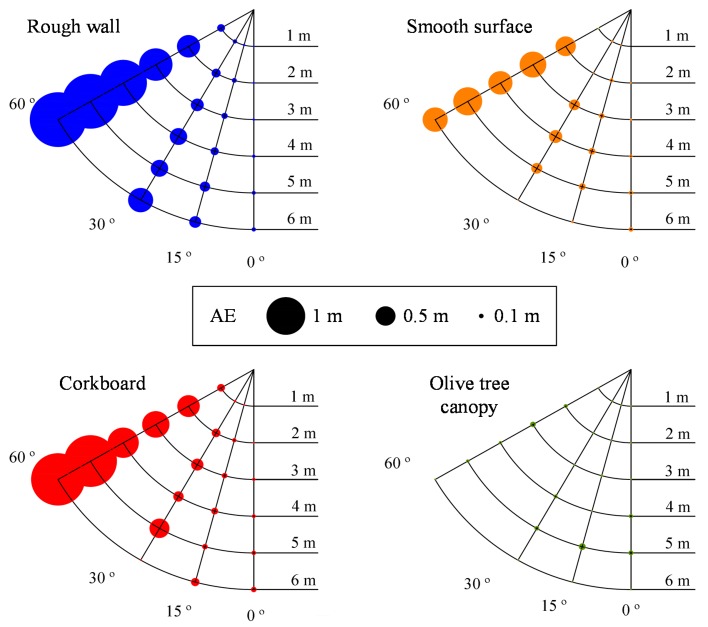
Mean absolute errors (AE) measured at different distances for the studied angles.

Figure 6 shows the mean absolute errors for various tested materials and distances. Except for the olive tree canopy, as shown in the figure, this error is clearly dependent on the reflection angle of the ultrasonic wave on the target (Figure 2), with a higher importance in the rough wall.

Table 3 summarizes the relationship between the distance measured by the sensor and the real distance for different materials and angles. The relationships are very significant for all materials. γ_1_, γ_2_, and γ_3_ are always significantly different from zero (*p* < 10^−4^), even though sometimes the differences between angles for the same material are not important in the proposal use of these sensors. There is no homogeneous behavior of the materials in that sense. Thus, the error results are low up to 30° for the corkboard or smooth board and then abruptly increase at 60°, whereas the reading error for the rough wall increases continuously with the increase in angle. Finally, it should be noted that the reading errors for the olive tree are small (less than 1%, Table 3, Figure 6) for every angle. This results could show, indirectly, the effect of absorption of sonic energy in the olive tree target that, in this case, is favorable to reduce the errors of sensor measurements. Nevertheless, the mean errors range from 14.4% to 27.5% for 60° for the rest of the materials.

The trial's purpose was to know if the ultrasonic sensor, widely tested in hedgerow orchards where the angle of incidence of the sonic wave on the canopy is always perpendicular to the row direction, has a significant loss in accuracy due to the difference in incidence angle. According to the results, ultrasonic sensors are suitable for measuring distances and, therefore, volumes in isolated trees because of the low errors observed in the test. Even though the angle of incidence seems to have a very strong effect on the accuracy of the sensor's measurements for the rest of materials, it seems to have a totally different behavior when working with olive canopies. A possible explanation for this phenomenon could be that, the entire canopy produces the same response, independent of the orientation of the most external facing part of the crown, because leaves are arranged at varying angles. The lower errors compared to those found for other targets could be explained by the fact that olive vegetation absorbs a significant part of the energy of the incident sound wave. This fact supports the results obtained for the sound cone, where the olive tree had the lowest cone diameter. This circumstance could have an important influence as well.

### 3.4. Interference Test

**Figure 7.**
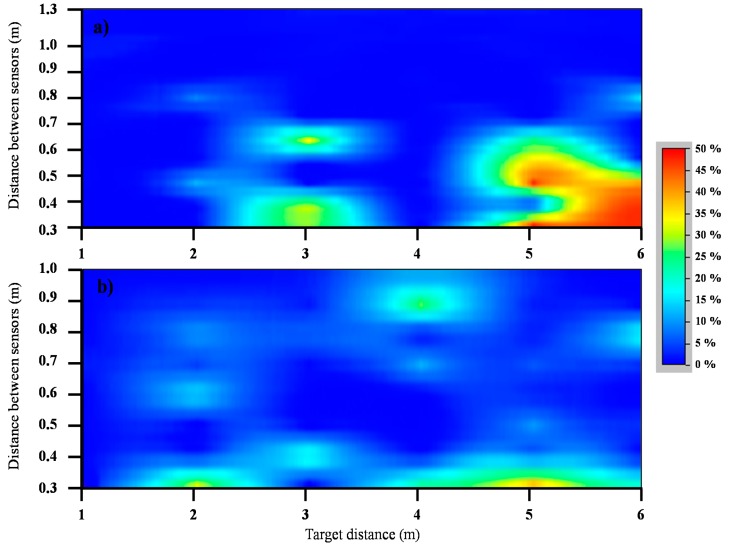
Percentage error due to sensor crosstalk as a function of the sensor spacing (Y axis) and target distance (X axis) for (**a**) an olive tree target and (**b**) a rough wall target.

The results obtained from the interference trial (Table 4, Figure 7) indicate that significant interference generally does not appear when the sensor separation is equal or higher than 1 m. The readings for the rough wall at a 4-m distance are an exception, with errors of 8.7% with a 1 m sensor spacing and errors up to 26% with a 0.9-m sensor spacing. These atypical results can be easily observed in Figure 7 because they appear in different colors and they were observed during the trial and do not have an obvious explanation. Independent of the normal error obtained for a certain target distance and sensor spacing, there were situations in which the sensors exhibited strange behavior. This is thought to occur because of the coupling between the emitted waves of different sensors that appear randomly.

Figure 7 show the different behaviors of both materials. For the olive target (Figure 7a), the errors are located at the lowest sensor spacing, especially at 5- and 6-m target distances, and constant results with low errors were observed for the largest separations. On the other hand, for the wall, the obtained errors exhibit more scattered behavior. It should be noted that the maximum percentage error was higher for the olive tree (47.2%) than the wall (37.4%). Similarly, the results show that interference errors do not appear for any sensor spacing for short distances (1 m), where the sound cone is still narrow. This result supports the work of Escolà *et al.* [23], who found that interference appeared at long distances within the sensor's reading range, and a sensor spacing of 60 cm was sufficient to avoid important interference errors in all measurement ranges.

The measured sonic cone seems to have a strong effect on the interference between sensors. Distances in the range from 3 to 5 m, where the maximum of the cone occurs (Figure 5), have the maximum interference effect in most cases (Table 4). This result is very logical if the interference effect is considered to be a reflection of the next sensor's wave; a wider cone results in a larger amount of interference. For the olive tree, the errors for a sensor spacing of 1.3 m are low, reaching a worst-case absolute error of 7.1 cm (at 5 m). In other studies [19], a sensor spacing of 1.89 m has been used; this was possible because they synchronized the sensors' readings to avoid interference problems. According to the results of the present study, that sensor spacing should be more than enough to prevent sensor interference, even when using long-range sensors similar to those studied in the present paper. On the other hand, it is not possible to determine the minimum sensor spacing to avoid unexpected coupling problems; thus, aforementioned atypical results could appear, even if it does not seem to be likely.

As coupling problems do not seem to be easy to avoid, sensor triggering could be an interesting alternative to study by repeating readings with different sensor spacings and determining the minimum distance to ensure that these problems will not appear. As the tested sensor presents an input pin that allows the user to send a square signal that would trigger measurements, different possibilities could be tested.

**Figure 8.**
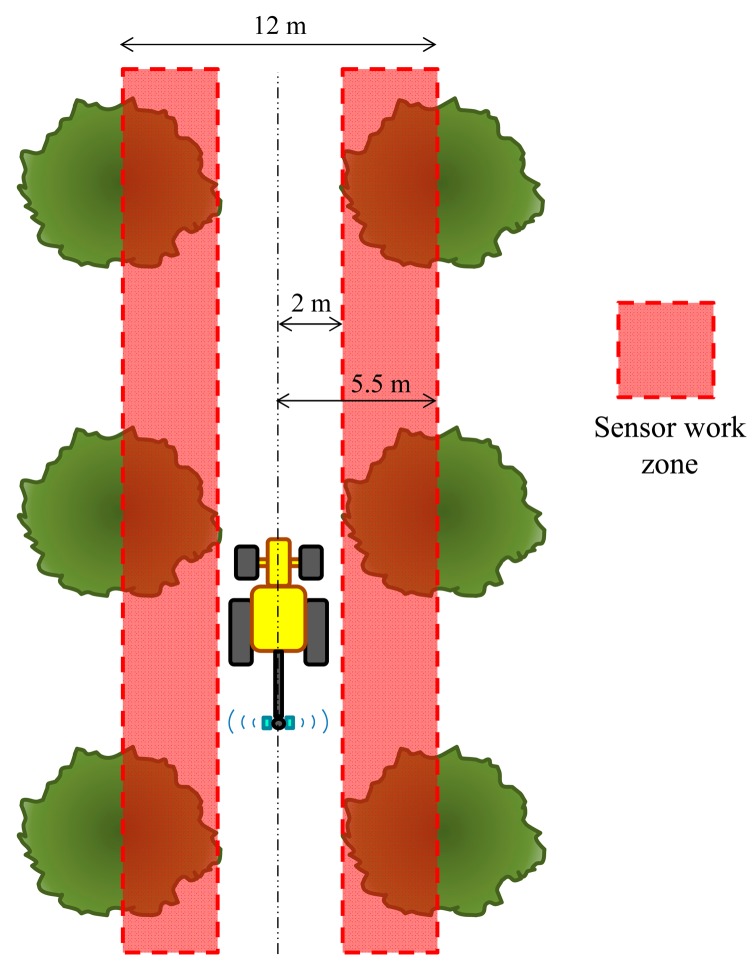
Schematic of the main working distance of the ultrasonic sensor for a 12-m olive tree spacing.

According to the most usual distances present in traditional olive tree crops, the sensors would operate between 2 and 5.5 m of reading distance, with a very important fraction of time working between 2 and 3.5 m (Figure 8). Therefore, interference errors should only be important in a relatively small part of the entire canopy, *i.e.*, in the most external parts of the trees.

### 3.5. Static Field Test

**Figure 9.**
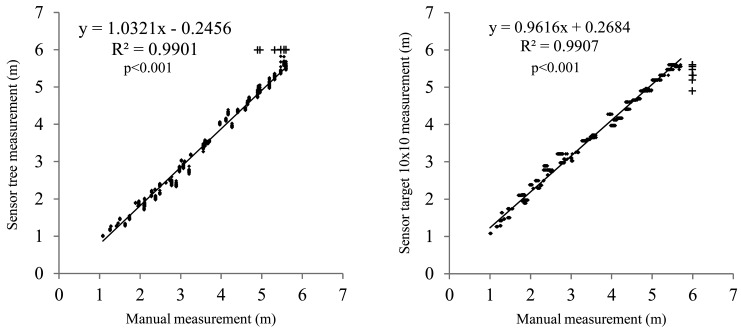
Relationship between the real and ultrasonically measured distances to olive canopies: (**a**) without and (**b**) with the 100 × 100 m^2^ square target.

Measurements with and without a target were almost identical (Figure 9), except for the smallest distances, where the ultrasonic sensor's readings for the target were slightly smaller than those measured directly on the canopy. This could be due to the existence of gaps, which are not recognizable by the sensor when the distance is long because of cone widening but are important for small distances. This was already described in other studies [24,26].

Similarly, Figure 9 shows a point accumulation at a distance of 6 m, representing the fact that the sensor is not able to obtain data at distances greater than 6 m. As the distances in the field were randomly selected, some of them exceeded the maximum measuring range of the sensor. According to the different tests performed, the ultrasonic sensor evaluated in this study is appropriate for measuring distances in olive tree plantations. The main drawback obtained from the study is that there is an important effect due to interference between sensors. Different sensor separations were tested to identify the minimum distance to avoid the crosstalk effect, but atypical results found seem to be difficult to avoid by only increasing the sensor spacing. Even though other problems could be present, synchronizing the sensor readings seems to be an interesting solution to explore in further studies.

## 4. Conclusions

Different tests were performed to evaluate the capability of an ultrasonic sensor to measure the distances to olive tree canopies, its accuracy, and its main problems. The following conclusions can be drawn:

- Even though the sound cone becomes very wide for certain distances, the olive tree canopy produces the narrowest sound cone among all studied materials, with positive results for practical applications in the field.
- The angle and distance have a marked effect on the sensor's accuracy by reducing it. Fortunately, the errors are greatly reduced for the olive tree canopy compared to the other tested materials, most likely owing to its higher absorption of sound waves and the irregular arrangement of leaves.
- The interference between sensors is very significant for small sensor spacings and long distances. The errors are very important up to a sensor spacing of approximately 0.8 m and are nearly zero when the sensor spacing is 1.3 m. This spacing of 1.3 m is practical under field conditions if the dimensions of traditional olive trees are taken into account.
- Sensor triggering should be tested because of the random error appearing as a consequence of the wave coupling phenomenon between sensors.
- The readings under field conditions are very accurate for all measurement ranges. The calibration curve fitted in the laboratory conditions was appropriate for field conditions as well. The future user can use this calibration curve to measure distances in olive tree after adjusting all the internal parameters of the sensor following the instructions given by the manufacturer.
- Generally, the evaluated sensor could be considered as appropriate for adjusting spray volume in the olive tree canopies, as the evaluated parameters had little influence on the sensor accuracy, and low errors were observed compared to the width of the measurement range of the sensor. This fact means that the spray volumes calculated according to the ultrasonic sensor measurements will be accurate enough to improve the actual spray applications in olive tree.

## Figures and Tables

**Table 1.** Main technical specifications of the Pepperl + Fuchs UC6000-30GM-IUR2-V15 sensor.

**Main Characteristics**	**Value/Range**
Sensing range	250–6000 mm
Adjustment range	400–6000 mm
Unusable area	0–350 mm
Transducer frequency	approx. 65 kHz
Response minimum delay	285 ms
Operating voltage	10–30 V DC
OUTPUT type	*Current output*: 4–20 mA
	*Voltage output*: 0–10 V
Resolution	≥0.35 mm
Work ambient temperature	−25 to 70 °C
Connection type	Connector M12 × 1,5-pin
Protection degree	IP65

**Table 2.** Main technical specifications of the CompactRIO-9025 real-time controller.

**Main Characteristics**	**Value/Range**
Operating System/Target	Real-Time
Controller Type	Rugged Performance
CPU Clock Frequency	800 MHz
System Memory	512 MB
Operating Temperature	–40 to 70 °C

**Reconfigurable Chassis**	**Value/Range**

Number of Slots	8
Specific FPGA	Virtex-5 LX110

**Analog Input Module**	**Value/Range**

Number of channels	8 analog input channels
ADC resolution	16 bits
Nominal input	0 to 20 mA

**Table 3.** Linear relationship between the measured distance [y] and the real distance [x] for the angle effect trial. For all materials, R^2^ = 0.99, *p* < 10^−4^, *n* = 720. Model *y_i_* = α*_0_ x* + ε*_i_*.

**Material**	**Angle**	**α_0_**
**Rough wall**	0°	0.985
	15°	0.951
	30°	0.898
	60°	0.725

**Smooth surface**	0°	0.984
	15°	0.975
	30°	0.961
	60°	0.856

**Corkboard**	0°	0.980
	15°	0.967
	30°	1.000
	60°	0.759

**Olive tree**	0°	1.010
	15°	1.007
	30°	1.008
	60°	0.994

**Table 4.** Mean absolute error and SE values for different sensor spacings and target distances. Values in bold indicate errors 5% above the real distance.

			**Target Distance (m)**					

			**1**	**2**	**3**	**4**	**5**	**6**
**Distance between Sensors (m)**	**Olive Tree Sample**	**1.3**	0.009 ± *0.001*	0.008 ± *0.002*	0.048 ± *0.001*	0.036 ± *0.009*	0.071 ± *0.022*	0.046 ± *0.013*
		**1**	0.004 ± *0.001*	0.005 ± *0.001*	0.006 ± *0.001*	0.047 ± *0.005*	0.006 ± *0.001*	0.005 ± *0.001*
		**0.9**	0.003 ± *0.001*	**0.163** ± *0.037*	0.004 ± *0.001*	0.004 ± *0.001*	0.006 ± *0.001*	**0.988** ± *0.170*
		**0.8**	0.004 ± *0.001*	0.003 ± *0.001*	0.005 ± *0.001*	0.003 ± *0.001*	0.004 ± *0.001*	0.007 ± *0.001*
		**0.7**	0.003 ± *0.001*	0.017 ± *0.014*	**0.983** ± *0.066*	0.005 ± *0.001*	**1.238** ± *0.187*	0.012 ± *0.002*
		**0.6**	0.005 ± *0.001*	0.003 ± *0.001*	0.003 ± *0.001*	0.004 ± *0.001*	**1.804** ± *0.150*	0.008 ± *0.001*
		**0.5**	0.004 ± *0.001*	**0.224** ± *0.035*	0.004 ± *0.001*	0.005 ± *0.001*	**2.288** ± *0.001*	**2.425** ± *0.155*
		**0.4**	0.004 ± *0.001*	0.004 ± *0.001*	**0.930** ± *0.099*	0.003 ± *0.001*	0.004 ± *0.001*	**2.801** ± *0.001*
		**0.3**	0.004 ± *0.001*	0.004 ± *0.001*	**0.856** ± *0.108*	0.005 ± *0.001*	**2.346** ± *0.001*	**2.833** ± *0.001*

	**Wall Sample**	**1**	0.006 ± *0.001*	0.019 ± *0.003*	0.061 ± *0.007*	**0.347** ± *0.074*	0.148 ± *0.043*	0.005 ± *0.001*
		**0.9**	0.005 ± *0.001*	0.070 ± *0.007*	0.063 ± *0.010*	**1.030** ± *0.005*	0.111 ± *0.020*	0.004 ± *0.001*
		**0.8**	0.003 ± *0.001*	**0.208** ± *0.029*	**0.186** ± *0.021*	0.062 ± *0.006*	0.083 ± *0.008*	**1.134** ± *0.163*
		**0.7**	0.021 ± *0.003*	0.092 ± *0.023*	0.034 ± *0.004*	**0.431** ± *0.104*	**0.274** ± *0.029*	0.180 ± *0.065*
		**0.6**	0.003 ± *0.001*	**0.297** ± *0.026*	0.005 ± *0.001*	0.009 ± *0.001*	0.121 ± *0.014*	0.005 ± *0.001*
		**0.5**	0.006 ± *0.001*	0.008 ± *0.001*	0.006 ± *0.001*	0.015 ± *0.002*	**0.463** ± *0.149*	0.196 ± *0.054*
		**0.4**	0.005 ± *0.001*	0.032 ± *0.003*	**0.595** ± *0.103*	0.036 ± *0.007*	**0.299** ± *0.029*	0.006 ± *0.001*
		**0.3**	0.006 ± *0.001*	**0.604** ± *0.014*	0.015 ± *0.001*	**0.898** ± *0.165*	**1.888** ± *0.127*	**1.154** ± *0.234*
